# A model with combined viral and metabolic factors effectively predicts HBeAg status under long term entecavir therapy: a prospective cohort study

**DOI:** 10.1186/s12985-015-0409-y

**Published:** 2015-11-02

**Authors:** Fen Liu, Feng Zou, Xiwei Wang, Huaidong Hu, Peng Hu, Hong Ren

**Affiliations:** Department of Infectious Diseases, Second Affiliated Hospital of Chongqing Medical University, Linjiang Road, Yuzhong District, Chongqing, China; Institute for Viral Hepatitis, Key Laboratory of Molecular Biology for Infectious Diseases, Ministry of Education, Chongqing Medical University, Linjiang Road, Yuzhong District, Chongqing, China

**Keywords:** Chronic hepatitis B, Entecavir, Long-term, HBeAg, Seroconversion

## Abstract

**Background & Aim:**

The aim was to extract factors from virologic and biochemical profiles at baseline and 24 weeks of treatment to predict HBeAg seroconversion in patients treated with ETV.

**Methods:**

HBeAg positive chronic hepatitis B patients receiving ETV naïve-treatment were enrolled. HBV DNA, ALT, and serological markers were prospectively monitored every 6 months for 240 weeks. The cumulative rates of virologic response (VR), biochemical response (BR), and HBeAg seroconversion were determined, and potential predictors for HBeAg seroconversion were identified through uni/multivariate analysis.

**Result:**

Two hundred twenty nine patients were eligible for this study. The cumulative rates of VR, BR, and HBeAg seroconversion at 240 weeks were 88.4 %, 100 %, and 36.7 %, respectively. Multivariate analysis showed that HBV DNA (OR, 2.8, *p* = 0.003), ALT (OR, 2.6, *p* = 0.005) at baseline, undetectable HBV DNA within 24 weeks (OR = 3.2, *p* < 0.001), and body mass index (BMI) ≥24kg/m^2^ (OR = 0.038, *p* = 0.013) were associated with HBeAg seroconversion. A prediction model for probability of HBeAg seroconversion was constructed. Patients can be classified into high (>40 %), intermediate (20–40 %), or low (≤20 %) groups based on the calculated probability of HBeAg seroconversion. The cumulative rates of HBeAg seroconversion were different among the three groups (*p* < 0.001). About 58 % patients in the high probability group achieved HBeAg seroconversion while almost 90 % patients within the low group remained HBeAg positive.

**Conclusion:**

A combination of HBV DNA, ALT and BMI values at baseline, and undetectable HBV DNA level within 24 weeks can predict HBeAg seroconversion. Both viral and metabolic factors likely determine HBeAg status with ETV treatment.

**Trial registration:**

CTR20132358

## Background

Chronic hepatitis B virus (HBV) infection currently affects approximately 350–400 million people worldwide [1]. It is well established that patients with high serum HBV DNA levels are associated with worse prognosis than those with low or undetectable HBV DNA [2]. Therefore, a major goal of clinical management of chronic HBV infection is to prevent progression of the liver injury and inflammation through sustained suppression of HBV replication [3–5].

Currently, treatment mainly relies on long term use of nucleos(t)ide analogues (NAs). NAs, especially new generation NAs including entecavir (ETV) can significantly inhibit viral replication to undetectable level in more than 80 % of treated patients, and normalize ALT in almost all of them following long term therapy [6]. However, HBeAg seroconversion facilitated by ETV treatment remains low with variable frequencies [7, 8].

HBeAg seroconversion is a critical event in natural course of chronic HBV infection and during the antiviral treatment [9]. It coincides with naturally reduced replication or sustained suppression of viral replication by antivirals, and is linked with reduced risk for progression of liver injury [10]. Patients who have successfully experienced this switch usually become so-called inactive HBsAg carrier who shows low serum HBV DNA and normal ALT. Importantly, HBeAg seroconversion is accompanied by significantly reduced HBV covalently closed circular DNA (cccDNA) level in the liver [11]. Reduced cccDNA level signals a better prospect for maintaining low level of HBV replication [12]. If patients stay at HBeAg positive phase, even though the liver inflammation has been brought under control, they may face a higher risk for quick restoration of high level of HBV replication because of relatively high intrahepatic cccDNA level once the antiviral therapy ceased [13]. Therefore, HBeAg seroconversion is an important endpoint achieving a better outcome of antiviral therapy.

So far, several baseline and on-treatment predictors for HBeAg seroconversion have been suggested in the literature. Medium range of HBV DNA, higher ALT level, and lower serum HBsAg level are considered baseline predictors [14–16] for HBeAg seroconversion [17, 18]. In addition to viral factors, we reasoned that NAs mediated inhibition takes place within infected cells and efficient access to viral replication site in each of infected cells by the drug is essential for efficient inhibition function. Such access could be impeded if there is excessive accumulation of biologic molecules that clog intracellular traffic in hepatocytes. It is well-known that hepatic steatosis is increasingly prevalent. For instance, about 30 % of CHB patients concur with hepatic steatosis [19]. We hypothesized that metabolic factors can impact antiviral response and HBeAg seroconversion.

The aim of this study was to evaluate efficacy leading to HBeAg seroconversion associated with long term ETV treatment and to build a model with the combined viral and metabolic factors and to assess predictive utility of the model for predicting HBeAg seroconversion.

## Results

### Study population

**Fig. 1:**
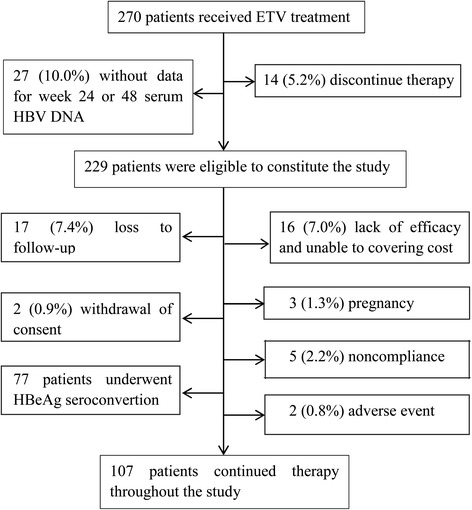
The flow chart showing enrollment and exits of patients during the course of treatment

Of enrolled 270 CHB patients with HBeAg positive, Forty-one patients, 14 (5.2 %) showed nonresponse at week 48 and they discontinued the ETV therapy, and 27 (10.0 %) had unavailable serum HBV DNA data at week 24 or 48, were excluded (Fig. 1). A total of 229 patients were eligible for this study. Table 1 showed the baseline characteristics of study patients. Mean age was 30.7 ± 7.6 years old. There were 177 (77 %) male patients and 23 (10.0 %) patients had cirrhosis. The median baseline serum ALT level was 134.2 ± 93.5 IU/L and HBV DNA level was 8.1 ± 1.3 log_10_copies/ml. The median treatment duration was 212 weeks (range 84 to 337 weeks). During the follow-up visits, 77 patients who reached the primary endpoint with HBeAg seroconversion exited from the treatment, and additional 45 patients ceased treatment. Reasons for discontinuation included lack of efficacy and the financial ability to cover high cost (16, 7.0 %), loss to follow-up (17, 7.4 %), pregnancy (3, 1.3 %), withdrawal of consent (2, 0.9 %), noncompliance (5, 2.2 %), and adverse event (2, 0.9 %). Finally, 107 patients continued therapy throughout the study.

**Table 1:** Baseline characteristics of enrolled patients

Characteristics	HBeAg positive
	(*n* = 229)
Age,yr	30.7 ± 7.6
Male,(%)	177(77 %)
BMI (kg/m^2^)	22.3 ± 2.4
Cirrhosis	23(10 %)
Median treatment duration	212(84–337)
Median HBV DNA(log10copies/ml)	8.1 ± 1.3
Serum ALT (IU/L)	134.2 ± 93.5
Serum AST (IU/L)	81.2 ± 54.0
Serum ALP (IU/L)	103.1 ± 32.6
Total bilirubin (mg/dL)	0.9 ± 0.3
Serum albumin (g/L)	45.2 ± 3.5
Platelet count (10^9/L)	153.1 ± 80.0
Serum creatinine (mg/dl)	0.9 ± 0.2
WBC (10^9/L)	5.4 ± 1.4

### Antiviral efficacy of long term ETV therapy

**Fig. 2:**
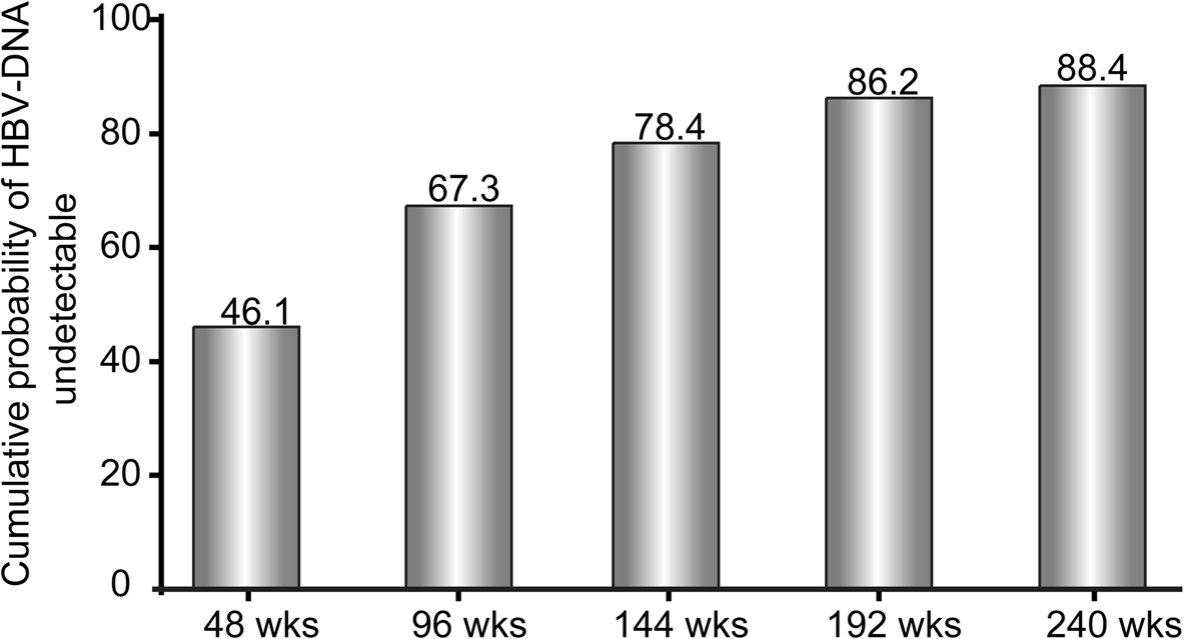
The cumulative probability of undetectable HBV DNA through the observation duration by Kaplan–Meier analysis

**Fig. 3:**
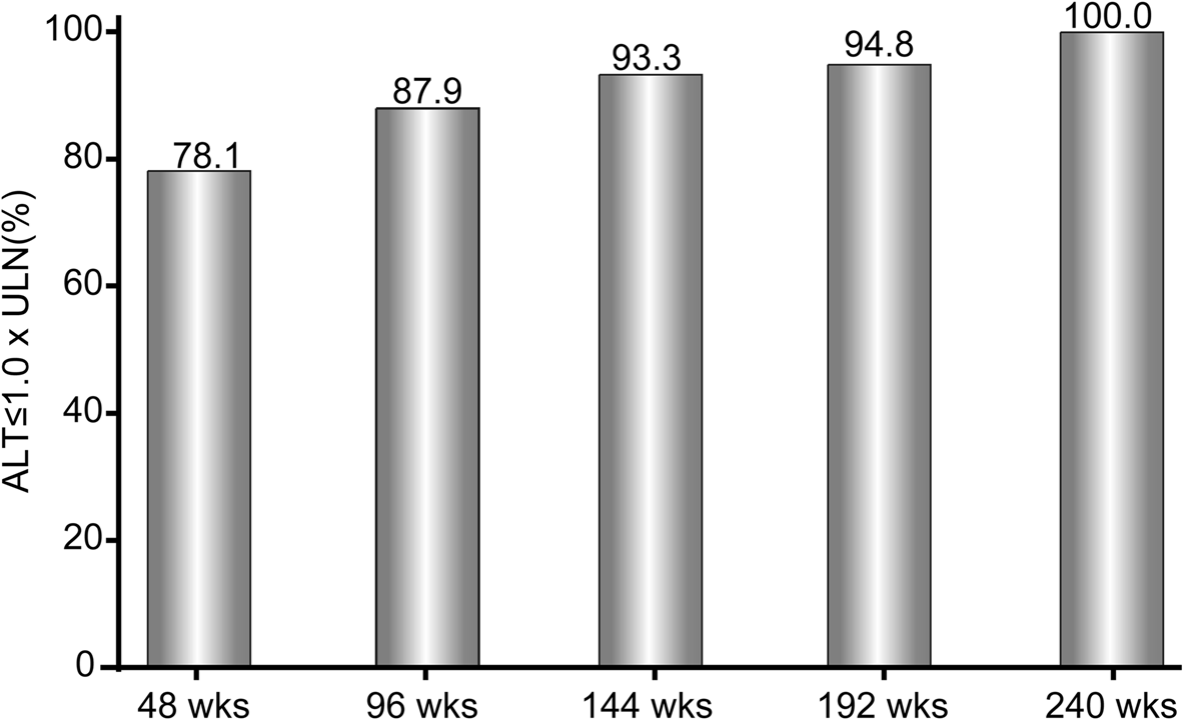
The cumulative probability of ALT normalization through the observation duration by Kaplan–Meier analysis

**Fig. 4:**
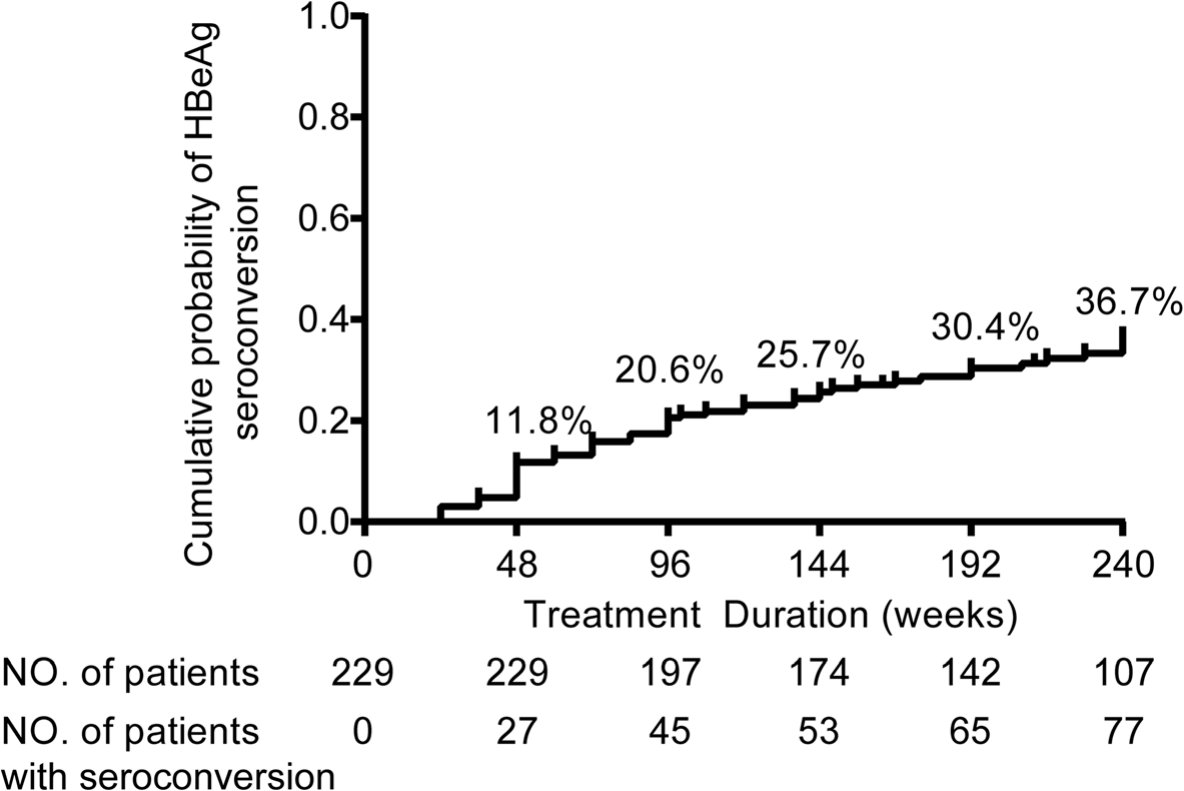
The cumulative probability of HBeAg seroconversion by Kaplan–Meier analysis

The cumulative probability of undetectable HBV DNA increased from 46.1 % at 48 weeks to 88.4 % at 240 weeks (Fig. 2). Proportion of ALT normalization was incrementally expanded as well. Up to 240 weeks, nearly all people had reached normal range (Fig. 3). During the study, 77 patients underwent HBeAg seroconversion. Two patients achieved HBsAg loss. The cumulative rates of HBeAg seroconversion were 11.8 %, 20.6 %, 25.7 %, 30.4 %, and 36.7 % over the duration of 1, 2, 3, 4, and 5 years, respectively, as displayed by Fig. 4. Seven patients experienced virological breakthrough; 3 of them were accompanied by biochemical breakthrough. Six patients still had detectable HBV DNA at the last visit. All of them showed good compliance. The patients with virologic breakthrough or detectable HBV DNA were tested for the genotypic mutations. Two patients were documented to confer resistance to ETV; the amino acid substitution loci were + L180M + T184G + S202I + M204V and M204V + L180M + S202G + V173L, respectively. The two received an ADV- add-on regimen. Among the patients without drug resistance, 8 patients switched to ETV plus ADV combination therapy, and three increased ETV dosage to 1mg per day.

### Comparison of pretreatment and on-treatment factors between patients with and without HBeAg seroconversion

Demographic and clinical data at baseline and on-treatment in patients with and without HBeAg seroconversion were summarized in Table 2. Mean age (≤or > 40 years) and gender ratio were well-balanced between two groups. The baseline HBV DNA level was stratified to three subgroups: 1. ≤7; 2. 7–9; and 3. ≥9 log_10_ copies/ml. A majority of patients with HBV DNA at 7–9 log_10_ copies/ml showed HBeAg seroconversion while HBeAg seroconversion in patients with high HBV DNA load >9 log_10_ copies/ml occurred much less frequent and the majority remained HBeAg positive. The difference was statistically significant (*P* = 0.001). All patients were also divided to two groups based on the baseline ALT level at 200IU/L. The percentage of patients with ALT ≥200 IU/L with HBeAg seroconverted was nearly two times higher than that of the non-seroconverted (*P* = 0.008). Interestingly, patients with HBeAg seroconversion had lower baseline BMI (categorized as normal BMI <24kg/m^2^, overweight BMI ≥24 kg/m^2^ based on the World Health Organization guidelines for adult Chinese population [20]) than that of the non-seroconverted group (mean, 21.6 ± 2.3 vs. 22.6 ± 2.5, *p* = 0.004), and the percentage of patients with BMI < 24kg/m^2^ in the former group was significant higher than that of the latter group (90 % vs.67 %, *p* < 0.007).

**Table 2:** Comparison of clinical features between patients with and without seroconversion

Characteristics	Seroconversion	Non-seroconversion	*p*-value
	(*n* = 77)	(*n* = 152)	
Age yr			
Age ≤ 40	67 (34 %)	132 (66 %)	0.971
Age > 40	10 (33 %)	20 (67 %)	
Gender			
Female	19 (36 %)	33 (64 %)	0.613
Male	58 (33 %)	119 (67 %)	
BMI (kg/m2)			
<24	69(40 %)	102(60 %)	<0.001
≥24	8(14 %)	50(86 %)	
HBV DNA level(log10copies/ml)			
HBV DAN < 7	10(21 %)	38(79 %)	0.001
7 ≤ HBV DNA < 9	56(44 %)	71(56 %)	
HBV DNA ≥ 9	11(20 %)	43(80 %)	
Baseline ALT level (IU/L)			
ALT ≥ 200	25(49 %)	26(51 %)	0.008
ALT < 200	52 (29 %)	126 (71 %)	
Undetectable HBV DNA within 24 weeks	32 (54 %)	27 (46 %)	<0.001
Undetectable HBV DNA within 48 weeks	44 (40 %)	65 (60 %)	0.062
Normalization ALT within 12 weeks	12 (50 %)	12 (50 %)	0.232
Normalization ALT within 24 weeks	25 (56 %)	20 (44 %)	0.001
Reduction HBV DNA at 24 weeks from baseline	4.7 ± 1.2	4.0 ± 1.4	<0.001
Reduction HBV DNA at 48 weeks from baseline	5.0 ± 1.0	4.9 ± 1.3	0.609
Reduction ALT at 12 weeks from baseline	106.0 ± 109.0	61.1 ± 102.3	0.002
Reduction ALT at 24 weeks from baseline	123.6 ± 106.7	77.0 ± 91.1	0.001

Clear differences in reduction of HBV DNA to undetectable level within 24 weeks from baseline (*P* = 0.014), and reduction of ALT to normal range within 12 weeks (*P* = 0.002) or 24 weeks (*P* = 0.001) from baseline were observed between two groups. However, if each parameter was used independently to predict the probability of HBeAg seroconversion, the corresponding AUS value was low (ranging from 0.5 to 0.7), and the positive predictive value was not promising.

There was no correlation between BMI and HBV DNA or ALT at the baseline (*P* = 0.098 and 0.071, respectively). Notably, BMI was negatively correlated with the decline of HBV DNA level within 24 weeks, and the correlation coefficient was −0.7; no correlation between BMI and other factors was found.

### Predictive factors associated with HBeAg seroconversion

To avoid confounding effects, we adjusted the on-treatment factors, and included the following in the regression analysis: the time of undetectable HBV DNA (≤24 weeks, between 24 and 48 weeks, >48 weeks), and the time of the normalization of ALT(≤12weeks, between 12 and 24 weeks, >24weeks). Our univariate analysis showed that BMI, HBV DNA and ALT levels at baseline, the time of VR, and the time of the normalization of ALT were the factors associated with HBeAg seroconversion (Table 3). However, the multivariate Cox proportional hazards analysis showed that BMI with more than 24kg/m^2^ (odds ratio [OR], 0.038; 95 % confidence interval [CI], 0.2 to 0.8; *p* = 0.013), baseline HBV DNA level <9 log_10_copies/ml (OR, 2.8; 95 % CI, 1.4 to 5.6; *p* = 0.003), baseline ALT level ≥ 200 IU/L (OR, 2.5; 95 % CI, 1.3 to 4.8; *p* = 0.005), and undetectable serum HBV DNA within 24 (OR, 3.2; 95 % CI, 1.9 to 5.5; *p* < 0.001) were significantly important in predicting HBeAg seroconversion. Differed from other variables, BMI ≥24 kg/m^2^ was negatively related to HBeAg seroconversion.

**Table 3:** Analysis of baseline factors for HBeAg seroconversion by multivariate cox proportional hazard analysis

Baseline characteristics	Univariate	Multivariate		
	P	Odd ritio	95 % CI	P
Age, yr	0.438			
Gender (male/female)	0.896			
BMI (≥24 kg/m^2^)	<0.001	0.4	0.2–0.8	0.013
Baseline ALT (IU/L)				0.004
ALT < 80	0.003	1	1	
80 ≤ ALT < 200	0.995	1.1	0.6–2.0	0.83
ALT ≥ 200	0.002	2.5	1.3–4.8	0.005
Baseline HBV DNA(log10copies/ml)				<0.01
HBV DAN < 7	<0.001	1	1	
7 ≤ HBV DNA < 9	<0.001	2.8	1.4–5.6	0.003
HBV DNA ≥ 9	<0.001	0.8	0.3–2.0	0.773
Time of undetectable HBV DNA				<0.01
≤24 weeks	<0.001	3.2	1.9–5.5	<0.001
24 < HBV DNA ≤ 48 weeks	<0.001	1.3	0.6–2.9	0.485
>48 weeks	0.385	1	1	
Time of Normalization ALT				
≤12 weeks	0.346			
12 < ALT ≤ 24 weeks	0.018			
>24 weeks	0.007	1	1	

### A model to predict HBeAg Seroconversion

A model for predicting HBeAg seroconversion was constructed using regression coefficient for each covariate obtained through multivariable logistic regression analysis. The Hosmer-Lemeshow goodness-of-fit test was good (*P* = 0.915). The regression formula for predictive probability of HBeAg seroconversion (P) is:

*P* = e^A^/(e^A^ + 1), and A = −1.549 + 0.792(if baseline ALT level ≥ 200 IU/L IU/L)-0.309 (if ALT level (<200) + 1.290 (if baseline HBV DNA level ≤ 9 log_10_ copies/ml)-0.403(if HBV DNA level >9) -1.388 (if BMI ≥24kg/m^2^) + 1.188 (if undetectable HBV DNA within 24 weeks)-0.364 (if undetectable HBV DNA within 48 week).

**Fig. 5:**
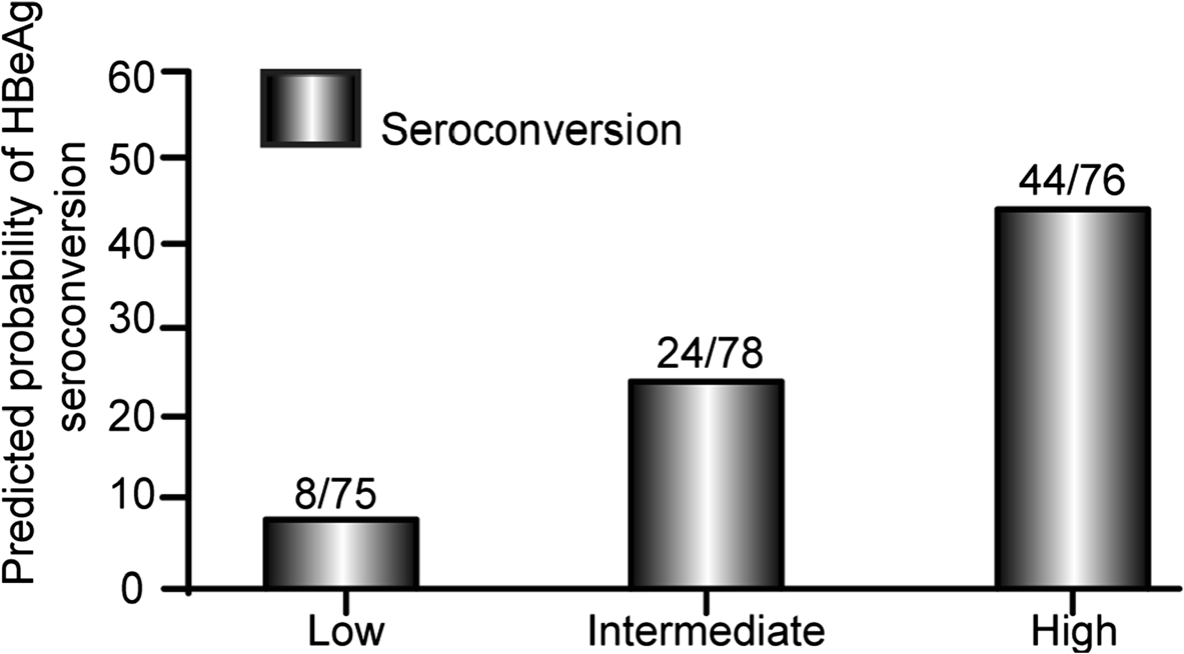
Distribution of patients with HBeAg seroconversion among three groups stratified by probability calculated by the model

**Fig. 6:**
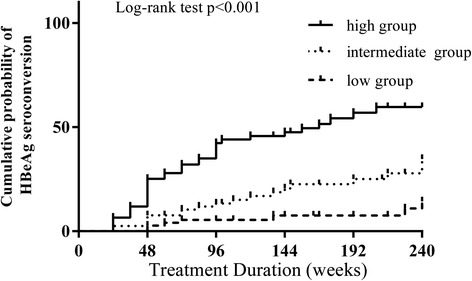
The cumulative probability of HBeAg seroconversion determined by the model

The probability for HBeAg seroconversion in each of all patients was computed and determined, and then two cut-off values were designated (see the justification for each value selection in Discussion). According to the calculated individual probability and the cut-off values, patients were categorized to high (>40 %), intermediate (20 %-40 %), or low (≤20 %) HBeAg seroconversion group. Approximately 58 % of patients in the high probability group achieved HBeAg seroconversion compared with 11 % and 31 % in the low and intermediate probability groups, respectively (Fig. 5). The seroconversion frequencies were significantly different (p < 0.001). The cumulative rates of HBeAg seroconversion in the high response group were significant greater than another two groups at the same time points. The cumulative rate increased slowly and remained nearly unchanged throughout the study period in the low probability group (Fig. 6, log rank test *p* < 0.001).

## Discussion

ETV is one of new generation NAs that have demonstrated more potent inhibition of HBV DNA replication than earlier ones. However, HBeAg seroconversion facilitated by ETV therapy remains relative low, for instance only about 20 % of HBeAg positive patients achieved HBeAg seroconversion after 48 weeks ETV therapy [21]. Factors associated with HBeAg seroconversion are not well understood and it is difficult to predict HBeAg seroconversion. But an ability for physicians to accurately predict HBeAg seroconversion is needed to improve clinical management of patients with chronic hepatitis B. We investigated the antiviral efficacy of long term ETV treatment of HBeAg positive chronic HBV infected patients in this study. We found that ETV was a highly potent antiviral agent. The cumulative rates of VR and BR were comparable to those observed in previous studies [22, 23]. We focused on identifying and testing both viral and metabolic factors, which were extracted from virologic and biochemical profiles at baseline and within 24 weeks of treatment, for impacting HBeAg seroconversion. We found that BMI values were inversely related to changes in HBeAg status, and serum HBV DNA, ALT values at baseline, as well undetectable HBV DNA level within 24 weeks after the treatment were positively related to HBeAg seroconversion. We constructed a model that incorporated both viral and metabolic factors, and can assign each patient with low, or medium or high probability for serum HBeAg seroconversion. The cumulative rate of HBeAg seroconversion at the end of 5 year treatment in this study was lower than previous results [7, 24]. Possible factors included that the dominant HBV strain in China is genotype C, which is associated with lower rates of seroconversion than genotypes A or B [25]. Additionally, the subjects who discontinued therapy were considered as failure that may underestimate the actual conversion rates.

An interesting factor identified by this study was an indicator for abnormal metabolism: BMI (≥24kg/m^2^) that was inversely associated with HBeAg seroconversion. In agreement with this finding, we noted that patients with higher BMI showed poorer virologic response at early phrase (before 24 weeks). The poorer virologic response suggests an inefficient inhibition of viral replication, which may have contributed to delaying HBeAg seroconversion. Some studies previously indicated that BMI was an independent factor for hepatic steatosis [26]. And hepatic steatosis was documented to have a negative effect on the therapy efficacy, and even it could result in ETV treatment failure [27]. Although conclusive data on the effect of steatosis remains to be established, a hypothesis is that not only viral factors, but also metabolic factors like hepatic steatosis in CHB patients are likely determining antiviral response including HBeAg seroconversion. It is possible that the contact between the HBV replication site and drug within hepatocytes could be separated or blocked by accumulated fat [28]. As a result of such separation, the drug may not effectively access to the viral replicating site . This difficulty may be further impeded by diminished activity of hepatic cytochromes in steatosis hepatocytes [19]. Additionally, patients with hepatitis C that is often marked by hepatic steatosis, are frequently accompanied by obesity and insulin resistance that may result in dysfunction of cellular immunesystem [29]. Currently obesity is increasingly spreading among various populations even in developing countries, and lipid metabolism disorders such as hepatic steatosis occur more frequently among general population, It is not surprising that CHB patients can have hepatic steatosis and the prevalence for such co-existence is up to 30.5 % [30]. Further studies would be expected to focus on impacts of steatosis on antiviral response at molecular level, and it may call for new antiviral strategy for CHB patients with hepatic steatosis or higher BMI.

This study also revealed that undetectable HBV DNA within 24 weeks was the most important independent predictive factor for HBeAg seroconversion as its regression coefficient was the highest among the four predictive factors. The result is in line with previous study [31]. Furthermore,the finding could be explained by the suggestion that antiviral therapy with NAs can induce partial restoration of immune responses [32], which are necessary for the durable host-mediated control of infection. And it is thought that rapid HBV DNA suppression may reflect this restoration of immune response in the host by stimulating the T-cell response and enhancing the probability of reducing the HBeAg expression in the long term [33]. Therefore, HBV DNA level at 24 weeks is an essential marker to monitor HBeAg seroconversion in ETV treatment.

Apart from two factors mentioned above, relatively lower HBV DNA levels (<10^7^ copies/ml) at baseline were independently associated with a stepwise increasing rate for the HBeAg seroconversion. A same finding was observed in previous studies [34]. Consistently, HBeAg seroconversion in patients treated with LDT can be predicted by baseline HBV DNA and ALT levels [35]. ALT reflects liver injury/inflammation that could be triggered by the host immune response. Our result that a higher baseline ALT level (≥200 IU/L) was related to HBeAg seroconversion was similar to previous study by Tseng TC et al. [36]. Therefore, levels of baseline ALT can be considered as a critical factor for predicting antiviral response including HBeAg seroconversion before tailoring antiviral treatment.

After identifying these four independent predictive factors, we constructed a formula that could assist physicians to predict approximately probability of HBeAg seroconversion for individual patients. As tested by this study, nearly 60 % of patients in the high probability group achieved HBeAg seroconversion. Interestingly extended study only resulted in a moderate increase in HBeAg seroconversion in the intermediate group. For those patients close monitor of HBV DNA before 48 weeks would be important to decide whether they were having an optimal treatment. In comparison, the cumulative rate of HBeAg seroconversion showed no additional increase in the low response group over time, suggesting that those patients may retain HBeAg for a long period despite long term ETV therapy. Therefore, patients with probability more than 40 % could have a great chance to achieve HBeAg seroconversion. Additionally, patients with BMI (<24Kg/m^2^), plus lower HBV DNA (>7 < 9log_10_copies/ml) and higher ALT (≥200IU/L) and HBV DNA undetectable before 24 weeks had a greater probability to develop seroconversion. The remaining patients were less likely for HBeAg seroconversion. Particularly, patients with BMI (≥24Kg/m^2^), high HBV DNA (>9 log_10_copies/ml) levels at baseline and detectable HBV DNA at 48 weeks were candidates for discontinued ETV treatment.

The cut-off values for the stratification of individual probability were decided by considering the natural history of CHB and recognized efficacy of ETV long-term treatment. According to prior studies, the annual incidence of spontaneous HBeAg seroconversion is about 2–15 %, and thus 20 % was chosen as the lower cut-off value to exclude spontaneous HBeAg seroconversion [37]. The probability of HBeAg seroconversion under ETV five years’ therapy is approximately 40 % or more [8], thus this value was selected as the upper cut-off line.

However, the predictive factors included in this study are not exhaustive, as some known risk factors were omitted, instead of using several easily available parameters. The hepatic steatosis was not included because ultrasound based detection of steatosis shows lower sensitivity and specificity, which largely depends on the operators’ skill. Recently, quantitative HBeAg and HBsAg levels have been shown to have predictive value for HBeAg seroconversion in ETV-treated patients [18]. However, the accurate HBeAg and HBsAg values were not available in this study, so those markers could not be assessed. Likewise, core promoter or pre-core mutations might represent additional factors for loss of HBeAg expression [38], but they are not routinely monitored in clinic. Therefore, the predictive power of this model for HBeAg seroconversion can be augmented if all predictive factors are incorporated.

This study is associated with a few limitations. First, the sample size was relatively small and the age range of patients was narrow because the HBeAg positive patients tend to be younger. The conclusions should be confirmed by future studies with large cohorts from multicenter. Second, all of our patients were Asians; therefore, it should be cautious to see if results can be extrapolated to other ethnic or genotypic patients. Finally, a lack of validated group to verify the prospective model is one challenge for all similar studies.

## Conclusions

Long-term ETV treatment was effective. Baseline HBV DNA and ALT levels and undetectable HBV DNA within 24 weeks were positive markers while BMI value was a negative factor for HBeAg seroconversion. Our model can separate treated HBeAg positive patients into three categories with differentiated probabilities. We tested and found that HBeAg seroconversion can be as high as 60 % in high probability group. The model may have potential to be incorporated into a clinical rate-prediction instrument that could improve the antiviral efficacy through appropriate choice at baseline, which could result in timely adjustment of regimen for individual patients. However, the observation should be confirmed in a prospective and larger numbers of patients study.

## Materials and methods

### Study population

This was a prospective cohort study that consecutively enrolled patients who entered the multicenter trial studies (CTR20132358) in the Department of Infectious Diseases, the Second Affiliated Hospital of Chongqing Medical University between March 2006 and April 2013. To explore the potential predictors for HBeAg seroconversion, patients recruited in the study were a component of the full study protocol. Patients were included into this study if they were hepatitis B surface antigen (HBsAg) and HBeAg positive for at least 6 months, baseline HBV DNA level greater than 10^5^copies/mL and ALT 1.3 times more than the upper limit of normal (ULN). All of patients were older than age 16. All of them were nucleoside-naïve and received ETV 0.5mg/day for at least 48 weeks. Patients with antibodies positive against hepatitis C or D virus, or human immunodeficiency virus, or those with decompensatory liver cirrhosis (ascites, jaundice, gastrointestinal bleeding, or encephalopathy) and a history of liver transplantation and HCC were excluded.

### Study design

The baseline characteristics and laboratory data of enrolled patients were recorded by chart review. Clinical evaluation was performed to record some general characteristics, such as age, gender, BMI, duration of treatment. Laboratory variables included serologic markers of HBV, serum HBV DNA levels and serum biochemistry data such as levels of ALT,aspartate aminotransferase,alkaline phosphatase, total bilirubin,serum albumin, serum creatinine, and blood routine examination. Patients were followed every 6 months to assess HBV DNA and ALT levels, drug tolerability and compliance. Patients with HBeAg seroconversion were followed for at least 24 weeks. Genotypic resistance was analyzed in patients with virologic breakthrough or relapse, and new antiviral regimens were given at the discretion of investigators, which was omitted in the H20080798. Patients who were lost during the follow-up or continued ETV therapy without seroconversion for the entire study period were considered as HBeAg seroconversion failure.

### Assay methods

HBeAg and anti-HBe were detected by AxSYM microparticle enzyme immunoassay (Abbott Laboratories, Abbott Park, IL, USA). Serum HBV DNA was quantified by real-time polymerase chain reaction (PCR) assay using the COBAS Taq-Man HBV quantitative test (Roche Molecular Systems Inc., Branchburg, NJ, USA), which has a low limit of quantification of 1000 copies/ml. Biochemical data were measured using an auto-analyzer (Roche Analytics; Roche Professional Diagnostics, Penzberg, Germany). The upper limit of normal for serum ALT level is 40 IU/L. Genotypic resistance was performed by restriction fragment mass polymorphism (RFMP) analysis.

### Evaluation of treatment efficacy and definitions

The primary endpoint was the HBeAg seroconversion, defined as undetectable HBeAg and detection of HBe antibody followed beyond 24 weeks. The second endpoints used the BR defined as ALT returned to the normal range, and the undetectable HBV DNA, defined as HBV DNA decreased to less than 1000 copies/mL. Virologic breakthrough was defined as an increase in HBV DNA level of >1*log_10_copies/ml compared with the nadir. Genotypic resistance was defined as the appearance of viral mutations bearing amino acid substitutions in the reverse transcriptase region. Each cumulative probability for respective undetectable HBV DNA, normalized ALT and HBeAg, was used to determine ETV antiviral efficacy.

### Data analysis

HBV DNA levels were logarithmically transformed. Continuous variables were expressed as mean ± SD. Categorical data were analyzed using the chi-square test. The correlation analysis was conducted by Pearson coefficient. Cumulative probability was evaluated by Kaplan-Meier analysis. Cox regression analysis was used to explore the factors with serologic response. To assess predictive accuracy of related factor, receiver operating characteristic (ROC) curves were constructed and the area under the ROC curve (AUC) was calculated. Multivariable logistic regression analysis was used to estimate the β regression coefficient and then to construct a multivariable linear model. The regression analysis was performed with stepwise selection, using a P value greater than 0.05 for removal and less than 0.1 for entering. All statistical tests were two-sided, and *P* < 0.05 was considered statistically significant. IBM SPSS statistics version 20 (IBM Corporation, Armonk, NY) was used for statistical analysis.

## Abbreviations

ETV: Entecavir
CHB: Chronic hepatitis B
VR: virologic response
BR: Biochemical response
BMI: Body mass index
HCC: Hepatocellular carcinoma
ALT: Alanine aminotransferase
HBsAg: Hepatitis B surface antigen
ULN: Upper limit of normal
NAS: Nucleos(t)ide analogues
ROC: Receiver operating characteristic
AUC: The area under the ROC curve
